# Dendritic Spines in Depression: What We Learned from Animal Models

**DOI:** 10.1155/2016/8056370

**Published:** 2016-01-10

**Authors:** Hui Qiao, Ming-Xing Li, Chang Xu, Hui-Bin Chen, Shu-Cheng An, Xin-Ming Ma

**Affiliations:** ^1^College of Life Science, Shaanxi Normal University, Xi'an, Shaanxi 710062, China; ^2^Department of Neuroscience, University of Connecticut Health Center, Farmington, CT 06030, USA

## Abstract

Depression, a severe psychiatric disorder, has been studied for decades, but the underlying mechanisms still remain largely unknown. Depression is closely associated with alterations in dendritic spine morphology and spine density. Therefore, understanding dendritic spines is vital for uncovering the mechanisms underlying depression. Several chronic stress models, including chronic restraint stress (CRS), chronic unpredictable mild stress (CUMS), and chronic social defeat stress (CSDS), have been used to recapitulate depression-like behaviors in rodents and study the underlying mechanisms. In comparison with CRS, CUMS overcomes the stress habituation and has been widely used to model depression-like behaviors. CSDS is one of the most frequently used models for depression, but it is limited to the study of male mice. Generally, chronic stress causes dendritic atrophy and spine loss in the neurons of the hippocampus and prefrontal cortex. Meanwhile, neurons of the amygdala and nucleus accumbens exhibit an increase in spine density. These alterations induced by chronic stress are often accompanied by depression-like behaviors. However, the underlying mechanisms are poorly understood. This review summarizes our current understanding of the chronic stress-induced remodeling of dendritic spines in the hippocampus, prefrontal cortex, orbitofrontal cortex, amygdala, and nucleus accumbens and also discusses the putative underlying mechanisms.

## 1. Introduction

Depression, a severe psychiatric disorder [1, 2], affects up to 20% of the population in the US within their lifetime and is more prevalent in women than men [3–6]. Although depression has been studied for decades, its cellular and molecular mechanisms still remain largely unknown [7]. As many as 30–40% of patients with major depressive disorder have treatment-resistant depression which does not respond to currently available antidepressant therapies [8]. It is therefore important to identify the mechanisms underlying depression in order to develop effective therapeutic strategies.

Chronic stress, especially psychosocial stressors in humans, is one well-known risk factor for the development of depression [6, 9–13]. Enhancement of neuronal plasticity is essential for adaptive intracellular changes during the normal stress response, which promotes dendritic growth, new synapse formation, and facilitates neuronal protein synthesis in the face of an acute challenge. In addition, a successful stress response requires continuity of the response to ensure normal brain function and promote survival [9, 14, 15]. On the one hand, brief or moderate stressors actually enhance neural function in most cases, while severe or chronic stressors are detrimental and can disrupt the ability of the brain to maintain its normal stress response, eventually leading to depression [15–18]. Furthermore, it has been shown that significant but brief stressful events (acute stress) result in the differentiation of stem cells into new nerve cells that improve the mental performance of rats [19]. On the other hand, chronic stress increases the levels of the stress hormone glucocorticoid and suppresses the production of new neurons in the hippocampus. This response results in decreased dendritic spine density and synapse number and impaired memory [17, 20–24]. The relationship between stress and psychiatric diseases has been well established for 20 years in the clinic [25, 26]. Chronic stress paradigms in rodents, the classical animal model of depression, recapitulate many of the core behavioral features of depression and respond to antidepressant treatments [10, 23, 27]. However, the precise nature of relationships among the effects of chronic stress, the dysregulation of spine/synapse plasticity, and the molecular mechanisms of depression remain poorly understood [9]. This minireview summarizes our current understanding, obtained from animal models of chronic stress, of remodeling of dendritic spines in five regions of the brain during depression.

## 2. The Plasticity of Dendritic Spines

**Figure 1:**
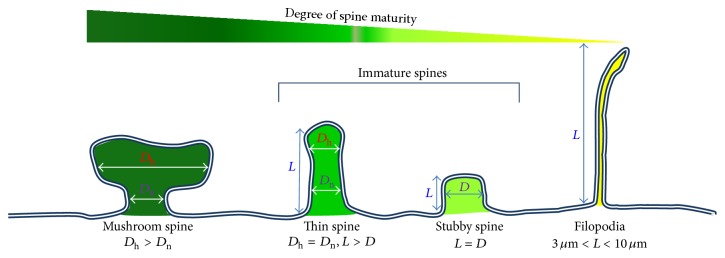
Diagram of dendritic spines. Dendritic spines are categorized into mushroom, thin, and stubby spines. Length of spine (*L*), diameter of spine head (*D*_h_), and diameter of spine neck (*D*_n_). Filopodia are the precursor of dendritic spine.

Dendritic spines are tiny membranous protrusions from the dendritic shaft of various types of neurons. They typically receive excitatory input from axons, although sometimes both inhibitory and excitatory connections are present on the same spine. Over 90% of all excitatory synapses that occur in the CNS are localized to dendritic spines [60], which are cellular substrates of brain connectivity and the major sites of information processing in the brain [61, 62]. Billions of neurons contact and communicate with each other via synapses. It is widely accepted that the regulation of dendritic spine number, size, and shape is of importance to the plasticity of synapses, as well as learning and memory [63, 64]. The morphology of spines is highly variable and commonly categorized into three types: thin, mushroom, and stubby (Figure 1) [65, 66]. Large mushroom spines are memory spines carrying more biochemical signals [67, 68] and a number of human disease states are associated with alterations with spine morphology and/or spine density [69]. Spines are thin if the length is greater than the neck diameter and the diameters of the head and neck are similar (Figure 1). Spines are classified as mushrooms if the diameter of the head is greater than the diameter of the neck. Spines are considered stubby if the length and width are equal. Spines are defined when they are no longer than three *μ*m [70]. The length of dendritic filopodia is normally >3 *μ*m and <10 *μ*m. The normal dendritic spine density ranges from 0.2 to 3.5 spines per 1 *μ*m of dendrite depending on the neuron type, age, and position along the dendrite as well as the method of counting [71]. Thin and stubby spines, as well as dendritic filopodia, are prevalent during development. Thin and stubby spines are considered to be immature, plastic spines. Dendritic filopodia are precursors of dendritic spines [60, 72]. The spine neck is an important structure for a mushroom spine to perform its normal function because the spine neck prevents Ca^2+^ exchange between the spine head and dendrite shaft. This is important for the regulation of synaptic transmission and may be neuroprotective, preventing excitotoxicity to the dendrite and neuron by restricting excessive influxes of Ca^2+^ within the synaptic region [69, 73]. Different spine types may serve different functions and changes in the ratio of these spines may have a greater effect on neuronal excitability and function [74]. It is generally accepted that thin spines are learning spines, whereas large, mature, and less motile mushroom spines harboring larger and stronger synapses are memory spines that are responsible for the maintenance of neuronal networks and long-term memory [75]. Large mushroom spines with large heads are stable and are likely to contain smooth endoplasmic reticulum, a spine apparatus, polyribosomes, and endosomal compartments in which posttranslational modification of proteins, local protein synthesis, local recycling of receptors, and membrane management occur, respectively [64]. Large mushroom spines that contain abundant AMPA receptors are not restricted to pairing with presynaptic axonal terminals containing more synaptic vesicles. They can also associate with presynaptic astroglial terminals, which enhance synapse formation, stabilization, and synapse elimination [64]. Mushroom spines with small heads are motile and unstable and contribute to weak or silent synaptic connections [68].

Dendritic spine pathology is associated with many psychiatric diseases [71, 76–78]. The formation, growth, and elimination of the dendritic spines are precisely controlled, which requires the reorganization of the neural network in response to acute stress or learning processes. These processes are commonly dysregulated or disrupted in chronically stressed animals [46, 79]. Therefore, understanding dendritic spines is fundamental in uncovering the mechanisms underlying depression. It is well established that depression is closely associated with selective structural changes, altered cellular resilience, and neuronal atrophy. Moreover, depression is associated with reduction in astrocytes and reduced/or increased volume of some brain regions that affect mood and cognition, which involve structural and molecular remodeling of dendritic spines in the hippocampus, prefrontal cortex, amygdala, and nucleus accumbens [7, 23, 49, 62, 80–83]. Antidepressants have reversed some of these structural changes observed in animal models of depression [13, 83, 84]. These studies have generated the hypothesis that alterations of the dendritic spines and the plasticity at excitatory synapses contribute to symptoms of depression [5, 85–88].

## 3. Chronic Stress and Animal Models of Depression

Animal models are essential tools for studying and understanding specific symptoms of human psychiatric disorders, though none of the current models fully recapitulate stress-related psychiatric disorders described in humans. Most of the current knowledge about the mechanism underlying depression has come from animal models. Several animal models of depression have been used to understand the mechanisms underlying depression [149]. We only discuss the model of chronic stress in this review. Several chronic stress models have been used to model depression-like behaviors in rodents such as chronic restraint stress (CRS), chronic unpredictable stress (CUS), and chronic social defeat stress (CSDS). Behavioral tests of anhedonia (sucrose preference) or despair (forced swim test and tail suspension test) have been widely used to determine depression-like behaviors induced by these three models [150]. Depression-like behaviors induced by these models can often be reversed by chronic antidepressant treatments [27, 86]. It is, however, worth noting that there are some rats or mice that do not respond to traditional antidepressants, which is similar to treatment-resistant depression in human subjects [151]. Here, we briefly summarize our current understanding about these three animal models.

### 3.1. Chronic Restraint Stress (CRS)

CRS has been used widely to study the morphological, hormonal, and behavioral alteration in several brain regions in rodents, such as the hippocampus, prefrontal cortex, amygdala, and nucleus accumbens because it is inexpensive and relatively easy to implement [152] (Tables 1–4). To study dendritic morphology and spine formation, this method typically involves restraining an animal for 1–6 h each day in a restraint device (bag or tube) for a period of 14–21 days or more. A disadvantage of the CRS model is the habituation of rats or mice to repeated exposure to homotypic restraint stressors; the response of plasma corticosterone, the major glucocorticoids in rodents, to the final stressor is diminished in animals that had been stressed for 14 days [153–156]. The pattern of hypothalamic corticotrophin-releasing hormone (CRH) heteronuclear RNA and mRNA responses to CRS is similar to the response of corticosterone, decreasing with increasing frequency of exposure to the repeated restraint stressor [153]. Animals habituate over time and finally show no increase in hypothalamic-pituitary-adrenal (HPA) axis activation and no increase in expression of hypothalamic CRH [30, 153, 156]. The duration of CRS may differentially affect learning/memory and CA3 dendritic atrophy with shorter periods of CRS (7–13 days) serving an adaptive function to enhance learning and memory [157]. On the other hand, longer CRS duration (21 days or more) causes maladaptive changes such as dendrite atrophy, spine loss, and impaired memory [15, 157, 158]. CRS-induced habitation of HPA axis contrasts with the hyperactivity of the HPA axis accompanied by increased CRH levels [43, 159] and the hypersecretion of cortisol [160, 161] in depressed patients, showing that activation of HPA axis is a hallmark of major depression [162, 163]. Depending on duration and intensity of chronic stress, some studies report that exposure of animals to CRS induces depression-like behaviors such as anhedonia (decreased sucrose preference) [164–169], which is a core symptom of human depression [10, 27]. A conflicting report shows CRS could not induce anhedonic-like behavior [170]. The duration and intensity of CRS as well as animal strains may determine whether CRS can be used as a valid animal model of depression to produce anhedonic-like behavior.

### 3.2. Chronic Unpredictable Mild Stress (CUMS)

CUMS is a well-established animal model for depression. The original, three-week chronic unpredictable severe stress (CUS) model with diverse severe and unpredictable stressors (electric shocks, immobilization, cold swimming, isolation housing, and other strong stimuli) was developed by Katz and coworkers [171, 172]. In order to accurately recapitulate the human condition, Willner and colleagues replaced severe stressors in Katz's model with mild stressors. Additionally, Willner and colleagues augmented the CUMS model with a variety of mild and unpredictable stressors (e.g., overnight illumination; presence of novel objects; periods of food and/or water deprivation; cage tilt; change of cage mate) [173]. In Willner's model, exposure of animals to 7–13 mild stressors up to 3 months produced a longer lasting depression-like behavior, anhedonia [173–175]. The CUS model used in Duman's group was modified from Willner's model. In Duman's model, animals were exposed to 10 [108, 176] or 12 [106] unpredictable stressors, 2 times per day, for up to 35 days, which produced depression-like behaviors. The duration of CUS is 21 days for the experiments using CUS alone or 35 days for the experiment using CUS together with antidepressant treatments [106, 108, 176]. It is worth noting that CUS model used by Duman's group is different from the CUMS protocol, not only in the duration and number of stressors/day, but also at the level of stressor intensity (rotation on a shaker 1 hour, cold 4°C 1 hour, lights off for 3 hours, lights on overnight, strobe light overnight, aversive odor overnight, 45° tilted cages overnight, food and water deprivation overnight, crowded housing overnight, and isolation housing overnight) [108, 176]. The modified CUMS model used in our laboratory consists of daily exposure of animals to 8 chronic unpredictable mild stressors, one stressor per day, for 21 days. The same stressor is not applied in two consecutive days [24, 177]. The different abbreviations of chronic unpredictable mild stress (CUS, CMS, or CUMS) were used in several modified versions by different laboratories. We use CUMS as a common denotation in this review. In comparison with the CRS model, CUMS overcomes stress habituation of the HPA axis occurring during stress, in which the response of plasma corticosterone to the final stressor is still sustained in animals which had been stressed for 15 to 35 days [27, 30, 106, 155]. Depression-like behaviors and deficits in synaptic plasticity are gradually developed during CUMS [24, 173]. The CUMS model recapitulates many of the core behavioral characteristics of human depression that are reversible by chronic treatments with traditional antidepressant agents [10, 27] and is more relevant to human disease. Therefore, the CUMS model has been widely used as an animal (specifically rat) model of depression. Our results show that, during CUMS, rats require three weeks to develop depression-like behaviors accompanied by both functional changes in CA3-CA1 synapses and decreased spine density in the dendrites of CA1 and CA3 pyramidal neurons [24, 177]. This is in line with Willner's CUMS paradigm [173], in which animals were exposed to initial unpredictable stress for three weeks to develop depression-like behaviors prior to the onset of antidepressant treatments. Because of its advantage of the gradual development of depression-like behaviors during CUMS [24, 175], this model is useful in studying depression-like behaviors such as anhedonia [27, 86, 174, 178]. In addition, this CUMS model is useful for inducing depression-like behaviors in female mice because chronic social defeat stress protocol cannot successfully induce depression-like behaviors in C57BL/6J female mice [179]. A recent report shows that C57BL/6 mice, one of the most widely used mouse strains, are resistant to the commonly used CUMS protocol due to the variety of genetically modified lines. A recently revised, eight-week CUMS protocol has been developed and used to induce depression-like behaviors in C57BL/6 mice [180]. Interestingly, male and female rodents are differentially affected by CUMS, depending on the behavioral and neurobiological markers that are being measured [181].

### 3.3. Chronic Social Defeat Stress (CSDS)

CSDS is one of the most frequently used rodent models for depression and has been used to induce depression-like behaviors in mice such as social avoidance and anhedonia [86, 144, 182–185]. During each defeat period, an intruder, a male C57BL/6J mouse, is allowed to interact for 10 minutes with an aggressive and large CD1 mouse during which the intruder is rapidly investigated, attacked, and defeated by the resident CD-1 mouse. The experimental C57BL/6J mice are exposed to a different resident aggressor for 10 minutes each day for 10 consecutive days [183, 184, 186–188]. On the one hand, after completing the social defeats, 30% of animals do not show depression-like behaviors known as “resilient,” a positive adaptation in the face of stress, threat, or severe adversity [189, 190]. On the other hand, a majority of animals (70%) develop depression-like behaviors known as “susceptible.” A disadvantage of this model is that it is limited in studying only male mice because female C57BL/6J mice are not easily defeated by CD-1 mice [86]. This model has been widely used to induce depression-like behaviors and study the molecular mechanisms underlying depression [139, 141, 142, 146, 149, 191, 192]. This model is also used to induce depression-like behaviors in rats [192, 193].

## 4. The Effects of Chronic Stress on Dendritic Spines in Different Brain Regions

### 4.1. Hippocampus (Table 1)

The hippocampus plays an important role in learning and memory and is particularly sensitive to stress and glucocorticoids [194, 195]. Rodent hippocampus contains high levels of glucocorticoid receptors (GRs) and mineralocorticoid-like receptors (MRs). The affinity of MR for corticosterone is 6- to 10-fold higher than that of GR, but it is GR that is activated after stress and is involved in its feedback action on stress-induced neural plasticity [196]. Chronic stress decreases GR expression or its numbers and finally alters the balance of GR/MR in the male hippocampus [197, 198], which is thought to be a protective mechanism against the damaging effects of chronic stress. Chronic exposure of male rats to glucocorticoids induces depression-like behaviors and causes the synaptic deficits in the hippocampus [58]. A recent report shows that GRs, acting via MR, decrease resilience to stress via downregulation of mGlu2 receptors in mice during CUMS [54]. Chronic stress and glucocorticoids impair hippocampal function, which in turn contributes to the HPA axis dysregulation [195, 198]. The blunting of the feedback mechanism is believed to underlie sustained high levels of glucocorticoids in some depressed patients [199]. People with depression have a significantly smaller hippocampus than healthy individuals [200–205], which may result from a decrease in dendritic arbors and spine density in hippocampal neurons. Hippocampal atrophy in depressed patients is associated with depression severity [206].

*CA1 and CA3 Dendrites.* Many structural and functional studies show that dendritic retraction or atrophy, characterized by both reduction in total dendritic length and a simplification of dendritic arbors, is found in the dendrites of CA3 pyramidal neurons but not the dendrites of CA1 pyramidal neurons in response to CUMS [49] or CRS [37, 38, 41, 42] (Table 1). Therefore, CA3 dendrites are more sensitive to chronic stress than CA1 dendrites. The different sensitivity of CA1 and CA3 to chronic stress may result from the differences between these two regions in afferents/efferents, the levels of GRs, NMDA receptors, 5-HT receptors, and GABA inhibitory tones [207–211]. GR levels are higher in the CA1 region than the CA3 region, where the receptors are activated by stress hormone corticosteroids [209, 212]. In addition, it has been repeatedly shown that apical dendrites of CA3 pyramidal neurons are more susceptible to the effects of sustained CRS than CA3 basal dendrites. Dendritic retraction in apical but not basal dendrites of CA3 pyramidal neurons is found after CUMS [49], chronic psychosocial stress [56, 57], and CRS [28, 30, 31, 33, 37, 38, 41, 42, 44, 49, 56, 213, 214]. CRS-induced depression-like behaviors and CA3 dendritic atrophy are not permanent but recovered to control levels after certain stress-free period following the end of CRS procedure [33, 49, 158, 213, 215]. Importantly, CA3 dendritic retraction induced by CRS requires corticosterone secretion and intact NMDAR function. Treatments of chronically stressed rats with either the steroid synthesis blocker cyanoketone or competitive NMDA receptor antagonist (CGP 43487) blocked CRS-induced dendritic retraction [31]. Similar to CUMS, rats usually require three weeks to develop depression-like behaviors and CA3 apical dendritic atrophy because only 21 days, but not 7 to 13 days of CRS, induces reversible impairments of spatial memory performance and CA3 apical dendritic atrophy [157, 158]. In addition, atrophy of apical dendrites, but not basal dendrites of CA3 pyramidal neurons, is found after chronic exposure to elevated glucocorticoid levels, which mimics chronic stress [216]. Chronic stress-induced hippocampal CA3 dendritic retraction and elevated glucocorticoid release contribute to impaired spatial memory [217].

*CA3 Dendritic Spines.* Chronic stress-induced alterations of spine density in CA3 pyramidal neurons depend on stressor types, animal species, sex, and the duration of stress. CRS causes either a decrease [30, 33, 36, 42, 44], an increase [29, 34], or no change [56] in the spine density in the dendrites of male rat CA3 pyramidal neurons. CRS-induced loss of synapses in male rat CA3 apical dendrites can be recovered following water maze training [34, 36]. One report shows that CRS causes a decrease in dendritic spine density, especially in thin and mushroom spines in mouse CA1 pyramidal neurons, but does not affect total spine density in mouse CA3 pyramidal neurons, due to increased stubby spine density and decreased thin and mushroom spine density [41]. The degree of stress-induced spine loss in CA3 pyramidal neurons correlates significantly with the memory defects and loss of LTP in mice [79]. In comparison with CRS, both 21-day CUMS and 30-day CUMS decrease spine density in male rat CA3 pyramidal neurons [24, 49], whereas 14-day CUMS increases spine density in male mouse CA3 pyramidal neurons [51], which is consistent with our report that two-week CUMS enhances LTP induction in CA3-CA1 synapses in male rat hippocampus [24]. Psychosocial stress (1 h/day for 28 days) does not affect spine density in CA3 pyramidal neurons of male tree shrews [56].

*CA1 Dendritic Spines*. CA1 is a hippocampal region crucial for long-term memory [218]. In comparison with CA3 pyramidal neurons, chronic stress-induced changes in spine density in CA1 pyramidal neurons are less characterized. Stress affects spine density in CA1 pyramidal neurons in a sex-dependent manner. Acute stress (30, 1 sec, 1 mA, 60 Hz shocks to the tail) increases spine density in the apical dendrites of male hippocampal CA1 pyramidal neurons but decreases it in the same area of female hippocampus [219]. These increases and decreases in spine density are dependent on NMDA receptor activation [35]. Similar to acute stress, the same CRS regimen causes a decrease in spine density in the apical dendrites of hippocampal CA1 pyramidal neurons in male rat and male mouse [40, 43, 46, 48] but causes an increase in spine density in the same region in female rats [37, 38, 42]. One recent study shows that CRS decreases spine density in basal dendrites, while it increases apical dendritic arbors in the CA1 pyramidal neurons of the ventral hippocampus in female but not in male rats [47]. In contrast to female rats, female mice show a decrease in spine density in CA1 pyramidal after exposure to same 21-day CRS [45]. Additionally, an ultrastructural study of CA1 synapses shows that 21-day CRS causes an increase in the size of the postsynaptic density in male rat CA1 [39]. Similar to CRS, CUMS also causes a decrease in spine density in the dendrites of CA1 pyramidal neurons in male rat [24]. Stress-induced increase in spine density in the apical dendrites of CA1 pyramidal neurons in female rat and same stress-induced decrease in spine density in the same area in male rat are completely prevented by NMDA receptor antagonist CPP [35, 219], but exposure of NMDA receptor antagonist CPP to the stress procedure does not affect corticosterone levels or the corticosterone response to stress, suggesting a key role of NMDA receptor activation in stress-induced increases or decreases in spine density [35]. Similar to sex-dependent alterations of dendritic spines induced by both acute stress and CRS in hippocampal CA1 pyramidal neurons, there is a sex difference in CRS-induced changes in hippocampal-dependent spatial learning and memory. CRS impairs spatial learning and memory in males but not in females [38, 197]. Furthermore, recent studies suggest that CUMS-induced glutamatergic dysfunction in excitatory temporoammonic- (TA-) CA1 synapses of the hippocampus serves as an underlying cause of depression [53, 220]. This suggests that restoring spine loss or excitatory synaptic dysfunction in the hippocampus could be a novel therapeutic target for depression. Similar to CUMS, chronic exposure of male rats to corticosterone for 3-4 weeks induces depression-like behaviors and causes a decrease in AMPAR-mediated excitation at temporoammonic-CA1 synapses accompanied by decreased expression of GluR1 protein. Blocking CUMS-induced increase of corticosterone during CUMS with the corticosterone synthesis inhibitor metyrapone prevents stress-induced depression-like behaviors [58]. Similar to male rats, exposure of male mice to 35-day corticosterone treatments shows anxiety/depression-like behaviors, accompanied by a reduction in spine density, mainly in thin and stubby spines but not in mushroom spines in CA1 pyramidal neurons [59]. Mushroom spines are more stable and resistant to corticosterone or CRS [46]. Chronic corticosterone-induced decreases in spine density in the hippocampal CA1 pyramidal neurons and depression-like behaviors recover to normal levels concomitantly after 25-day treatment with fluoxetine [59]. These studies suggest that corticosterone secreted during stress plays a key role in chronic stress-induced depression-like behaviors, dysfunction of excitatory synapses, and alteration of dendritic spines in the hippocampus; rescuing chronic stress-induced loss of dendritic spines and/or synaptic dysfunction may rescue depression-like behaviors.

### 4.2. Prefrontal Cortex (PFC) (Table 2)

The medial PFC (mPFC), an information processing center, is often divided into the anterior cingulate, prelimbic (PL), and infralimbic (IL) subregions. These subregions are different in structure and function [221]. The mPFC plays a critical role in the integration of cognitive and emotionally relevant information, modulation of subcortical systems, and attention [222–225]. The mPFC expressing high levels of glucocorticoid receptors [226] is a target site for glucocorticoids and plays an important role in the regulation of the response of HPA axis to stress and antidepressant response [225, 227, 228]. It is widely reported that the mPFC volume is decreased in a subset of depressed patients [201, 205, 229–233]. However, a recent report shows that the decreased volume of the mPFC is found in male but not in female depressed patients [234]. The decreased volume of the mPFC in depressed patients [201, 205, 229, 230] is in line with decreased expression of synaptic-function-related genes and loss of synapses in the mPFC of subjects with major depression disorder [110]. In addition, glial cell loss, reductions in the density and size of neurons in the postmortem mPFC of subjects with major depression, may contribute to pathology of depression [235, 236]. Animal studies show that the retraction of apical dendrites of pyramidal neurons in the mPFC induced by chronic stress is accompanied by alterations in fear conditioning and extinction [112]. CRS-induced dendritic retraction and spine loss in the hippocampal and mPFC neurons are accompanied by cognitive impairments, which are mediated by each respective structural alteration [92, 109, 217].

It is well documented that CRS results in a retraction of the distal part of apical dendritic arbors of layers II/III pyramidal cells [76, 89, 90, 92] and a decrease in spine density on those neurons [91, 93, 96, 237] in the mPFC of male rats, which is similar to that found in hippocampal CA3 region [41, 42, 44]. The pattern of CRS-induced dendritic reorganization is similar to that seen after daily corticosterone injections [238, 239]. CRS also alters spine morphology with an overall decrease in mean dendritic spine volume and surface area, a reduction in large mushroom spine density, and an increase in small thin spine density in the mPFC of male rats. These findings suggest failure of the spines to mature and stabilize following CRS [93]. One conflicting study, however, reports that CRS-induced decrease in spine density in the male rat mPFC is characterized by a decrease in thin and stubby spine density without affecting mushroom spine density [99].

CRS causes a reduction of length and branch number in the apical dendrites of the neurons in the mPFC of young (3 months) and aged (20 months) male rats. Surprisingly, CRS-induced retraction of apical dendrites, however, is reversed with recovery in young (3 months) but not aged (20 months) animals [98]. In young rats, CRS results in dendritic spine loss and alters the patterns of spine morphology. In contrast, CRS does not affect spine density and spine shape in aged animals, showing that dendritic spines become progressively less plastic in the aging brain [99]. Interestingly, chronic immobilization stress does not affect spine density in a subpopulation of IL neurons in the mPFC that project to the basolateral amygdala (BLA) in male rats, suggesting this pathway may be particularly resilient against the effects of stress [109]. Randomly selected neurons in the IL of the mPFC, however, show dendritic retraction after CRS. Since most layer II/III neurons project intracortically, the majority of randomly selected pyramidal neurons may be local cortical neurons with no projections to the BLA [109]. An independent study reports that IL neurons, but not PL neurons, in the mPFC are highly sensitive to a brief exposure to stress and the same form of stress impairs fear extinction in mice [112]. However, these IL neurons are putative local cortical neurons without projections to the BLA. A conflicting report shows that CRS causes dendritic retraction in PL neurons of rat mPFC, while this dendritic retraction is prevented by the D1R antagonist SCH23390, and the same D1R antagonist causes dendritic retraction in the PL neurons of the mPFC in unstressed rats. However, the effects of CRS on dendrites in the IL neurons of mPFC are not studied in this report [104]. These results show that dopaminergic transmission in the PL neurons of the mPFC during stress may contribute directly to the CRS-induced retraction of apical dendrites, while dopamine transmission in the absence of stress is important in maintaining normal dendritic morphology [104]. Recent reports show that acute foot-shock stress not only produces an increase in the number of excitatory synapses and docked vesicles [240] in the mPFC, but also induces rapid and sustained increases in spine density accompanied by atrophy of apical dendrites in the PL neurons of the mPFC [241]. Importantly, these synaptic changes induced by acute stress are prevented by chronic antidepressant desipramine treatments [240, 241]. Optogenetic activation of the mPFC exerts potent antidepressant-like effects, showing that the activity of the mPFC may play a key role in the development of depression-like behaviors and antidepressant responses [242]. Similar to hippocampus, alteration of stress-mediated dendritic arbors in the mPFC is sex dependent. CRS causes retraction of apical dendrite arbors in the mPFC in male, while it increases apical dendrite arbors in the female mPFC in which CRS-induced dendritic plasticity is estrogen dependent [97]. Rat mPFC is sexually dimorphic, which is characterized by a bigger and more complex apical dendritic tree in the PL neurons of the mPFC in healthy male rats than that in healthy female rats [243, 244].

### 4.3. Orbitofrontal Cortex (OFC)

The OFC, a part of the PFC in the frontal lobes in the brain, is involved in cognitive functions, decision-making, and emotional processing [245]. The studies from neuroimaging and neuropathology show that the OFC is involved in pathophysiology of major depression [246]. Decreases in cortical thickness, neuronal size, neuronal density, and glia densities in the II–IV cortical layers of the OFC are found in subjects with major depression [236]. The decrease in neuronal sizes in layer 3 of the OFC from depressed subjects is confirmed by another postmortem study [247]. Neuroimaging and functional studies also show that patients with major depression have reduced OFC volume [248] and reduced density of pyramidal neurons in layers V and III of the OFC [249]. In contrast, animal studies show that 3-week CUMS increases both the volume of layers II/III in the lateral orbital subregion and the volume of layer II in the ventral orbital subregion of the OFC, which is accompanied by an increase in the length of apical dendrites in the ventral orbital subregion of the OFC [107]. Interestingly, CRS causes a 43% increase in the dendritic arbors in the OFC neurons, an effect opposite to what is observed in the mPFC neurons where the same CRS causes 20% retraction of apical dendritic arbors in layer II/III pyramidal neurons of the mPFC [92]. The mechanisms through which CRS increases dendritic arbors of the OFC are not known. Further studies are needed to explore the discrepancy between the data from imaging analysis or postmortem studies and the findings from animal models. Our recent study showed that 3-week CUMS caused a decrease in spine density in the OFC pyramidal neurons, which was accompanied by both depression-like behaviors and decreased expression of Kalirin-7 and PSD95 in the OFC (Chang Xu, Shu-Chen An, and Xin-Ming Ma, unpublished). Kalirin-7 plays an essential role in maintaining dendritic spine density, size, and synaptic functions [250, 251]. Expression of Kalirin-7 in the hippocampus is decreased by 3-week CUMS [24]. Similar to CUMS, chronic exposure of male mice to corticosterone for 20 days that recapitulates blood corticosterone levels found after CRS exposure in mice also decreases spine density in the OFC neurons, which fails to recover after one week of washout period [130]. This suggests that chronic stress-induced decrease in spine density is not reversible in the OFC neurons. Additional study is required to address this question.

### 4.4. Amygdala (Table 3)

The amygdala, a structure within the subcortical limbic system, is involved in the processing of emotion and motivation such as fear and anger. The amygdala is also responsible for determining what memories are stored and where they are stored. There are conflicting reports on amygdala volume in major depression [252]. Imaging studies show an increase [253–255] or decrease [256, 257] or no change [258] in amygdala volume or increased activity of amygdala [201, 259, 260] in patients with major depression. A conflicting MRI study reports a trend towards smaller left amygdala volumes in depressed patients compared with healthy controls [203]. A postmortem study shows that depressed subjects have a larger lateral nucleus and a greater number of total BLA neurovascular cells than controls. There are no differences in the number or density of neurons or glia between depressed and control subjects [252]. To our knowledge, it is not clear whether cell size in BLA is altered in depressed patients.

Animal studies show that chronic stress generally results in an increase in spine density and enhanced dendritic arborization in the amygdala (Table 3). This is in contrast to the hippocampus and PFC (Tables 1 and 2). Acute immobilization also causes an increase in spine density without any effects on dendritic arbors in BLA spiny neurons [127], showing that these neurons are very sensitive to stress. Amygdala-dependent fear learning is enhanced by CRS in rats [33]. Chronic stress causes an increase in dendritic arborization and spine density in the BLA spiny neurons of male rats [122, 123, 125–129] and male mice [114, 118, 120, 124]. These neurons are thought to be glutamatergic neurons [261]. In contrast, CRS causes a decrease in spine density in spiny neurons in the medial amygdale, which are GABAergic neurons [114]. CRS-induced increase in dendritic arbors and spine density in the BLA pyramidal neurons and CRS-induced depression-like behavior in wild-type mice are absent in fatty acid amide hydrolase (FAAH) deficient mice [124] suggesting a key role of FAAH in maintaining normal amygdala function in the face of chronic stress. Chronic immobilization stress-induced dendritic hypertrophy in the BLA spiny neurons is not reversible [126]. This is distinct from hippocampal CA3 and mPFC atrophy, which is reversible within the same period of stress-free recovery [33]. A single dose of corticosterone induces dendritic hypertrophy in the BLA accompanied by enhanced anxiety [262]. Chronic exposure of mice to corticosterone for 20 days mimicking chronic stress increases dendritic length and spine density in the BLA [130]. Chronic exposure of rats to corticosterone for 2 weeks causes an increase in the levels of memory-related genes including Arc/Arg3.1 and Egr-1 and enhances the consolidation of fear memory processes in the lateral amygdala [131]. In addition, tianeptine, an antidepressant, exerts the opposite roles in chronic stress-induced synaptic changes in the amygdala and hippocampus [120].

### 4.5. Nucleus Accumbens (NAc) (Table 4)

Animal studies indicate that the neuronal circuitry of the PFC-NAc-ventral tegmental area (VTA) underlies drug reward responses and contributes to relapse to cocaine seeking [263, 264]. Excitatory axonal terminals from glutamatergic neurons of the PFC form the synapse onto NAc medium spiny neurons (MSNs), which also receive dopaminergic (DA) inputs from the VTA. The VTA receives GABAergic inputs from the NAc and glutamatergic inputs from the PFC [265, 266]. In addition, The NAc also receives glutamatergic inputs from ventral hippocampus and basolateral amygdala [146]. The NAc serves as a hub of the brain's reward pathways [267] and plays a central role in mood and emotion regulation [268]. Depressive symptoms, such as anhedonia, and depression severity are correlated with reduced NAc volume and reduced NAc responses to rewards in depressed patients [205, 269]. An optogenetic study shows that inhibition of the VTA-NAc projection induces resilience, whereas inhibition of the VTA-mPFC projection enhances susceptibility [270], highlighting a key role of PFC-NAc-VTA circuitry in the development of depression. Therefore, dysregulation of PFC-NAc-VTA reward circuitry may contribute to the pathophysiology of depression [13, 146, 271]. Similar to the effect of cocaine abuse, chronic stress may alter dendritic spines and synaptic plasticity in the PFC-NAc-VTA circuitry. A recent study, however, reports that chronic social defeat stress- (CSDS-) mediated increase in glutamatergic transmission at the intralaminar thalamus- (ILT-) NAc but not PFC-NAc circuitry mediates stress-induced postsynaptic plasticity on the MSNs and depression-like behaviors in susceptible mice [142].

The MSNs of dorsal striatum receive not only glutamatergic inputs from the cerebral cortex and the thalamus, but also DA innervation from the midbrain [272]. These MSNs account for >95% of the neurons in the striatum [273, 274]. The dorsal striatum and the NAc are not distinguishable in their populations and expression of DA receptors (DRs, D1R and D2R). Approximately half of the striatal MSNs express the D1R [274, 275]; other half MSNs express the D2R [276, 277]. The degree of D1R/D2R colocalization remains controversial, ranging from 10% to 30% [275, 278, 279]. D1R signaling enhances dendritic excitability and glutamatergic signaling in striatonigral MSNs, while D2R signaling exerts the opposite effect in striatopallidal MSNs (indirect pathway) [280–282]. CRS causes a decrease in AMPAR/NMDAR ratio in the D1R-MSN of the NAc compared to nonstressed control, while it does not affect AMPAR/NMDAR ratios in D2R-MSNs of the NAc. This CRS-induced decrease in the ratio of AMPAR/NMDAR in the D1R-MSN is accompanied by depression-like behaviors, showing a role of NAc D1R-MSNs, at least in part, in the development of depression [135]. This is further supported by two recent reports [143, 283]. One report shows that enhanced activity in D1R-MSNs causes resilient behaviors, while inhibition of these D1R-MSNs induces depression-like behaviors after CSDS [283]. Another report shows that CSDS specifically results in an increase in synaptic strength represented by the increased amplitude of uEPSCs (unitary excitatory postsynaptic currents) in large mushroom spines on D1R-MSNs but decreases synaptic strength on D2R-MSNS mushroom spines in the NAc of resilient mice. CSDS does not affect the uEPSC amplitude in small thin spines on both D1R- and D2R-NAc MSNs in resilient mice [143]. CSDS, however, does not alter synaptic strength in mushroom and thin spines on D1R- or D2R-MSNs in the NAc in susceptible mice [143]. These data show that the NAc D1R-MSN of susceptible mice may be resistant to adaptation and play a critical role in the development of chronic stress-induced depression-like behaviors. In addition, the inhibitor of kappaB kinase (I*κ*K) in the NAc is also a critical regulator of depression-like behavior, and the I*κ*K-nuclear factor kappaB (NF*κ*B) plays a key role in the regulation of synaptic signaling and neuronal morphology* in vitro* and* in vivo* [138]. Overexpression of I*κ*K increases thin spine density in the NAc MSNs. CSDS-induced increase in I*κ*K activity in the NAc enhances social avoidance behavior and promotes the formation of thin spines. Inhibition of I*κ*K signaling results in a reversal of CSDS-induced social avoidance behaviors, suggesting that CSDS-induced depression-like behaviors are associated with I*κ*K-mediated increase in thin spine density in the NAc [138]. Interestingly, CSDS-induced increases in stubby spine density and the frequency of mEPSCs in the NAc in susceptible mice are accompanied by an increase in the levels of I*κ*K in the NAc [139]. These results show that CSDS-induced increases in stubby spine density and I*κ*K expression in the NAc are correlated with depression-like behaviors. CSDS-mediated downregulation of Rac1 through an epigenetic mechanism contributes to depression-like behaviors and enhanced formation of stubby spines in the NAc MSNs of susceptible mice [141]. Furthermore, DeltaFosB, a transcription factor, plays an essential role in the mechanism of resilience in mice, supported by evidence that CSDS-mediated induction of DeltaFosB in the NAc is not only necessary and sufficient for resilience in mice, but also required for the antidepressant fluoxetine to reverse depression-like behaviors induced by CSDS [136]. NR2B in the NAc plays a key role in the modulation of CSDS-induced depression-like behaviors and synaptic plasticity. CSDS-induced reduction in NR2B surface expression in the mouse NAc neurons is restored by fluoxetine treatment. Behaviorally, restoration of NR2B loss prevents the behavioral sensitization of mice to chronic stress [137]. Overexpression of DNA methyltransferase (Dnmt3a) increases dendritic spine density in the NAc MSNs. CSDS-induced depression-like behaviors are accompanied by an increase in the Dnmt3a levels in the NAc, suggesting that CSDS-induced depression-like behaviors are positively correlated with increased spine density in the NAc neurons [140]. These studies highlight an important role of the NAc in chronic stress-induced depression-like behaviors. It is possible that stress may differently affect dendritic spines in the D1R-MSNs and D2R-MSNs of the NAc. More studies are required for a better understanding of the roles of D1R-MSNs and D2R-MSNs in chronic stress-induced depression-like behaviors and the underlying mechanisms.

Reduced NAc volume in depressed patients [205, 269] is not in line with the findings from animal models in which stress generally results in an increase in spine density in the NAc MSNs. CSDS causes an increase in spine density and the frequency of mEPSCs in the mouse NAc MSNs [86]. In addition, the shell of the NAc is thought to be a part of the extended amygdala [284]. Chronic stress increases spine density in the neurons of the BLA and the shell of NAc even though these two neuron types are naturally different. The downstream mechanisms of chronic stress-induced spine formation in these two distinct neuron types are not clear.

Taken together, these data show that altered spine density and synaptic plasticity in the NAc MSNs are correlated with depression-like behaviors induced by chronic stress, which may be a target for developing the novel treatment strategies for depression.

## 5. The Mechanisms of Chronic Stress-Induced Alterations in Dendritic Spines

The molecular mechanisms underlying spine loss and dendritic retraction induced by chronic stress in the hippocampus and PFC as well as enhanced spine formation found in the amygdala and NAc in chronically stressed animals are not well understood. Expression of several synapse-related genes is decreased in the postmortem PFC of subjects with major depressive disorder [110]. One of these genes is GATA1 (GATA-binding factor 1), a transcriptional repressor that plays a key role in the formation of dendritic spines and dendrite arbor maintenance [110]. Furthermore, a nuclear pore complex protein, nucleoporin p62 (NUP62), and tyrosine phosphorylation of NUP62 play a critical role in CRS-induced dendritic retraction of hippocampal CA3 pyramidal neurons [285]. Many synaptic proteins including Kalirin-7, spinophilin, Homer1, cofilin, Rac-1, cadherin, p-Akt, p-GSK-3*β*, p-Erk1/2, PKC, NCAM, PSA-NCAM, SNAP-25, SNAP-29, VAMP1/2, syntaxin 1A, synaptophysin, synapsin 1, Vglut2, GluR1, GluR2, NR1, NR2A, NR2B, PSD95, *α*CaMKII, melanocortin 4 receptors, CRH receptor 1, and P190RhoGAP play an important role in the regulation of the spine formation and/or synaptic plasticity; expression of these synaptic proteins in the brain is altered by chronic stress, and these proteins may play a key role in chronic stress-induced both depression-like behaviors and spine alterations (Table 1–4) [24, 40, 44, 46, 53, 100, 102, 113, 130, 141, 142, 144, 286–292]. In addition, chronic stress-induced alterations of several signal transduction pathways including cAMP-PKA-CREB, cAMP-ERK1/2-CREB, cAMP-PKA, Ras-ERK, PI3K-Akt, TNF*α*-Nf*κ*b, GSK-3*β*, mTOR, and CREB may be also associated with chronic stress-induced spine loss or increase in certain brain areas [7, 22, 293]. A recent report shows that the Homer1/mGluR5 complex is involved in the development of CSDS-induced depression-like behaviors [294], suggesting a role of this complex in chronic stress-mediated spine plasticity. Presynaptic mGlu2 receptors play a key role in CUMS-induced depression-like behaviors in male susceptible mice [54]. The rapid antidepressant-like properties of ketamine, an NMDA receptor antagonist, result from increased synaptic signaling proteins and increased number and function of new spine synapses via activating the mammalian target of rapamycin (mTOR) pathway in the rat mPFC and hippocampus [295–298]. S6K1, a key mediator of activity-dependent synaptic protein synthesis, is the downstream of mTORC1 and plays a critical role in CUMS-induced depression-like behaviors [299]. Postmortem studies show that the levels of NR2A, NR2B, mGLuR5, PSD-95, and mTOR as well as the levels of S6K, eIF4B, and p-eIF4B, the core downstream signaling targets of mTOR, are decreased in the PFC of depressed patients [300]. These studies suggest that mTOR signaling is a promising target for the development of novel antidepressant drugs [297, 301, 302].

Taken together, understanding chronic stress- and/or depression-induced alterations in dendritic spines, synapse plasticity, synaptic proteins, and their upstream/downstream signaling pathways may pave the path for developing efficiency therapeutic strategies for depression. The search for the mechanisms through which chronic stress alters dendritic spines or synapse numbers in different brain regions should be a major future direction.

## Figures and Tables

**Table 1:** The effects of chronic stress on dendritic spines in hippocampus.

#	Stress	Paradigms	Animals	CA1	CA3	References
1	CRS	6 h/day for 21 days	Male SD rats	nd	Apical, not basal dendritic atrophy	[28]

2	CRS	6 h/day for 21 days	Male SD rats	nd	↑ spine density in apical, basal dendrites	[29]

3	CRS or multiple stress (CMS): 3 different stressors	CRS, 6 h/day for 21 days CMS: 3 stressors/day for 21 days	Male SD rats	nd	Apical dendritic atrophy; CORT habituates to 21-day CRS but not 21-day CMS	[30]

4	CRS	6 h/day for 21 days	Male SD rats	nd	Apical dendritic atrophy is blocked by cyanoketone or CGP43487	[31]

5	CRS	6 h/day for 21 days	Male SD rats	nd	↑ synaptic vesicle density in MFT	[32]

6	CRS	6 h/day for 21 days	Male SD rats	nd	Apical dendritic atrophy, recovery after 10 days ↓ spine density	[33]

7	CRS	6 h/day for 21 days	Adult male Wister rats	nd	↑ excitatory MF-CA3 synapses, recovery after maze learning	[34]

8	Acute restraint plus intermittent tail shock	30 shocks: 1 mA, 1 s, 1/min	Adult male and female SD rats	↑ spine density in male and ↓ in female apical dendrites, both 100% blocked by CPP	nd	[35]

9	CRS	6 h/day for 21 days	Male Wistar rats	nd	↓ PSD number; ↓ spine density in apical dendrites Retraction of dendritic TE with ↓ in their volume	[36]

10	CRS	6 h/day for 21 days	Adult SD adult female rats	*↔* dendritic atrophy ↑ spine density ↑ spine size	Apical dendritic atrophy Spine density, nd	[37, 38]

11	CRS	6 h/day for 21 days	Male Wistar rats	↑ PSD surface and ↑ PSD volume; *↔* excitatory synapses in stratum	nd	[39]

12	CRS	6 h/day for 21 days	C57/BL6 male Wt mice	↓ spine density in apical dendrite ↓ NR1, NR2B, NR2A, and GAP43	These decreases are tPA and plasminogen dependent	[40]

13	CRS	6 h/day for 21 days	C57/BL6 male Wt mice	*↔* dendritic atrophy; ↓ total spine density, *↔* stubby spines ↓ thin and mushroom spine density	Apical, not basal dendritic atrophy *↔* total spine density, ↑ stubby spines, ↓ thin and mushroom spines	[41]

14	CRS	6 h/day for 21 days	Adult SD female rats	*↔* dendritic atrophy ↑ spine density ↑ mushroom spine	Apical dendritic atrophy ↓ spine density	[42]

15	CRS	2.5 h/day for 14 days	Male rats	↓ spine density in apical dendrites	nd	[43]

16	CRS	6 h/day for 21 days	Adult SD male rats	↑ spine density	Apical dendritic atrophy, ↓ spine density, and ↑ spinophilin and Homer1	[44]

17	CRS	6 h/day for 21 days	Female mice	↓ spine density in apical dendrites	nd	[45]

18	CRS	2.5 h/day for 14 days	Adult male SD rats	↓ spine density, ↓ cadherin, and *↔* LIMK/cofilin and p-LIMK/p-cofilin	nd	[46]

19	CRS	6 h/day for 25 days	Female, male Long-Evans rats	↓ spine density in basilar dendrites; ↑ apical dendritic arbors in female, not male ventral CA1	Deficits in spatial memory in female but not male	[47]

20	CRS	6 h/day for 21 days	Adult male mice	↓ spine density; ↓ p-Akt, ↓ p-GSK-3*β*, and ↓ p-Erk1/2	nd	[48]

21	CUMS	1 stressor/day for 30 days	Male Wister rats	*↔* apical dendrite	Apical dendritic atrophy; ↓ MF-CA3 synapses	[49]

22	CUMS	2 stressors/day for 10 days	Male Wister rats	nd	*↔* CA3 dendrites	[50]

23	CUMS	1 stressor/day for 21 days	Male SD rats	↓ CA1 spine density	↓ CA3 spine density, ↓ Kalirin-7 protein in hippocampus	[24]

24	CUMS	1 stressor/day for 14 days	Male mice	nd	↑ CA3 spine density	[51]

25	CUMS	1 stressor/day for 8 weeks	Male SD rat	↓ PSD thickness in CA1 ↓ PSD95 protein	↓ PSD93, ↓ PSD95, ↓ SYN, ↓ spinophilin, and ↓ synapsin 1	[52]

26	CUMS	2-3 stressors/day for 21–35 days	Adult SD rats	Impaired AMPAR-synaptic excitation at TA-CA1 synapses ↓ GluR1 and PSD95	Induces depression-like behaviors	[53]

27	CUMS	2 stressors/day for 28 days	Male C57/b mice	↓ mGlu2 receptors in susceptible, not resilient mice	mGLu2 deletion in mice results in a more severe susceptibility to stress	[54]

28	Multimodal stress	Adult male C57BL/6J mice	5 h	↓ synapse numbers in dorsal apical dendrites ↓ PSD-95-ir puncta	↓ synapse numbers in dorsal CA3 apical ↓ PSD-95-ir puncta	[55]

29	Psychosocial stress	1 h/day for 28 days	Male tree shrews	nd	Apical, not basal dendritic atrophy *↔* spine density	[56]

30	Psychosocial stress	1 h/day for 28 days	Male rats	nd	Apical dendritic atrophy	[57]

31	Chronic CORT exposure	3-4 weeks	Male SD rats	Impaired AMPAR-synaptic excitation at TA-CA1 synapses ↓ GluR1 protein	Induces depression-like behaviors	[58]

32	CORT exposure	35 days	C57/BL6 male mice	↓ CA1 thin and stubby spine density, but not mushroom spines	*↔* CA3 spine density	[59]

CRS: chronic restraint stress. CUMS: chronic unpredictable mild stress. TA: temporoammonic. CORT: corticosterone. MFT: mossy fiber terminals. TE: thorny excrescences in the stratum lucidum of CA3. *↔*: no change. ↓: decrease. ↑: increase. nd: not done.

**Table 2:** The effects of chronic stress on dendritic spines in the prefrontal cortex (PFC).

#	Stress	Paradigms	Animals	PFC	Proteins	References
1	CRS	6 h/day for 21 days	Male SD rats	↓ apical dendrite of layers II and III mPFC		[76]

2	CRS	3 h/day for 21 days	Male SD rats	Apical dendrite atrophy *↔* basal dendrites in PL mPFC		[89]

3	CRS	6 h/day for 21 days, 21 day recovery	Male SD rats	↓ apical dendrite length, reversible after 21 d in mPFC		[90]

4	CRS	6 h/day for 21 days	Male SD rats	↓ 20% apical dendritic length, ↓ spine density in PL mPFC		[91]

5	CRS	6 h/day for 21 days, 21-day recovery	Male SD rats	↓ 20% apical dendritic arbors in mPFC		[92]

6	CRS	6 h/day for 21 days	Male SD rats	↓ mushroom spine density ↑ thin spine number in PL mPFC		[93]

7	CRS	1 h/day for 7 days	Male SD rats	↓ spine density in PL mPFC		[94]

8	CRS	6 h/day for 21 days	Male SD rats	↓ apical spine density in apical dendrites Inhibition of PKC prevents spine loss		[95]

9	CRS	6 h/day for 21 days/with 21-day recovery	Male SD rats	↓ apical dendrite arbors, ↓ spine density; partial recovery of dendrites and spine loss in IL mPFC		[96]

10	CRS	3 h/day for 7 days	Male and female SD rats	↓ apical dendrite arbors in male, ↑ apical dendrite arbors in female, which is estradiol dependent in mPFC		[97]

11	CRS	6 h/day for 21 days	Male SD young and aged rats	↓ apical dendrite arbors in young, but not aged, rats are reversible; ↓ spine density in young, but not aged, rats		[98]

12	CRS	6 h/day for 21 days	Male SD rats young, middle-aged, and aged	↓ spine density (↓ thin and stubby spines, *↔* mushroom spines) in young but not middle-aged and aged rats in PL mPFC		[99]

13	CRS	6 h/day for 21 days	Male SD rats	↑ mRNA levels of VAMP2, VAMP1, syntaxin 1A, synapsin, synaptotagmins I and III, and synapsins I and II ↓ SNAP-25 mRNA level	↑ protein levels of VAMP2, syntaxin 1A, and SNAP-25	[100]

14	CRS	2 h/day for 7 days	Adult male WT mice	↓ spine density in mPFC; ↓ apical dendrites	↓ BDNF	[101]

15	CRS	1 h/day for 21 days	Male GIN mice	*↔* spine density in mPFC	↑ NCAM, SYN	[102]

16	CRS	6 h/day for 21 days	Male SD rats	↓ spine density in PL mPFC	Alpha-2A-adrenoceptor	[103]

17	CRS	3 h/day for 21 days	Male SD rats PL mPFC	↓ dendritic retraction is prevented by D1R antagonist SCH23390 that causes dendritic retraction in unstressed rats		[104]

18	CRS	2 h/day for 7 days	Male SD rats	↓ glutamatergic transmission in PFC pyramidal neurons		[105]

19	CUMS	15 days or 35 days	Male SD rats	35% ↓ cell proliferation in neocortex		[106]

20	CUMS	3 stressors/day for 21 days	Male Wistar rats	↓ volume of layer I/II of PL and IL ↓ neuronal density of layer II of PL and IL Apical dendritic atrophy in PL and IL *↔* spine density tends to decrease in PL and IL		[107]

21	CUMS	2 stressors/day for 21 days	Male SD rats	↓ spine density in mPFC; ↓ synapsin I, GluR1, and PSD95		[108]

22	CUMS	1 stressors/day for 21 days	Male SD rats	↓ synaptic length of the active zone in CG1 mPFC ↓ PSD thickness in PL; ↓ PSD93, ↓ PSD95, ↓ spinophilin in CG1 and PL	↓ spinophilin and synapsin 1 in CG1	[52]

23	CIS	2 h/day for 10 days	Male SD rats	*↔* apical dendrites in IL-BLA projecting neurons in IL mPFC Apical dendritic atrophy in random selected neurons in IL mPFC *↔* spine density in IL mPFC		[109]

24	Depressed patients		Postmortem dorsolateral PFC	↓ synapse number in dorsolateral PFC, ↓ synaptic-function-related genes	GATA1 ↑ Rab4b ↓	[110]

25	CORT, vehicle	daily injection for 21 days	Male SD rats	↑ spine density proximal to the soma		[111]

26	Forced swim	10 min/day for 3 days	Adult male C57BL/6J mice	↓ apical dendrites in IL mPFC *↔* basal dendrites in IL mPFC; *↔* apical and basal dendrites in PL mPFC		[112]

27	Early-life stress	3 h/day on postnatal days 1–14	Male Wistar rats	↓ spine density in apical and basal dendrites in mPFC	GluR1, GluR2, *α*CaMKII, and PSD95 ↑	[113]

CRS: chronic restraint stress. CUMS: chronic unpredictable mild stress. CIS: chronic immobilization stress. PL: prelimbic region of the mPFC. IL: infralimbic region of the mPFC. CG1: area 1 of cingulate region of mPFC. CORT: corticosterone. *↔*: no change. ↓: decrease. ↑: increase.

**Table 3:** The effects of chronic stress on dendritic spines in the amygdala.

#	Stress	Paradigms	Animals	Amygdala	Function	Proteins	References
1	CRS	6 h/day for 21 days	Wt C57/BL/6 mice	↓ spine density in WT medium spiny stellate neurons MeA, ↑ spine density in Wt BLA	*↔* spinogenesis in BLA OF tPA−/− mice	tPA−/− mice reverse stress-induced reduction of spine density in MeA	[114]

2	CRS	6 h/day for 28 days	Male young, Wistar rats	*↔* spine density in MePD			[115]

3	CRS	1 h/day for 10 days	Male ICR mice	↓ eIPSC, ↑ LTD GABAergic synapse in BLA	MAGL inhibition prevents depression-like behavior	2-AG ↑, MAGL ↓	[116, 117]

4	CRS	2 h/d for 10 days	Male Wt mice	↑ BLA dendritic branching ↑ spine density in BLA apical and basal dendrites ↑ spine length ↑ anxiety behavior	Fmr1 KO mice fail to show anxiety	In Fmr1 KO mice *↔* spine length in BLA ↓ spine density in BLA	[118]

5	CRS	1 h/day for 21 days	Male GIN mice	*↔* spine density, ↓ dendritic arborization in interneurons in LA and BLA		GAD67, synaptophysin and PSA-NCAM ↓	[119]

6	CRS	2 h/day for 10 days	Male ICR mice	↑ dendritic length and branch points in BLA, which are blocked by tianeptine	Depression-like behaviors are blocked by tianeptine	Tianeptine is an antidepressant	[120]

7	CRS	1 h/day for 14 days	Male SD rats	Impaired LTP in the NAc 30 days after stress termination	CB1/2R agonist prevents the stress-impaired LTP	↓ GRs in amygdala and NAc	[121]

8	CRS	20 min/day 7 out of 9 days	Male SD rats	↑ dendritic length in BLA, ↑ spine density in LA and BA, but proximal increase in LA, nonproximal increases in BA	↑ frequency of sEPSC *in vivo*		[122, 123]

9	CRS	6 h/day for 21 days	C57/Bl6 mice	↑ dendritic arborization ↑ spine density in BLA ↑ anxiety-like behaviors	CRS-induced changes in structure and behaviors are abolished in FAAH KO mice		[124]

10	Acute restraint stress CRS	Single 1 h	Male young adult	↓ Spine density in the posterodorsal MePD			[115]
		Single 6 h 6 h/day for 28 days		*↔* spine density in MePD			

11	CIS CUMS	2 h/d for 10 days 10 days	Male Wistar rats	↑ dendritic arborization in BLA pyramidal and stellate neurons Dendritic atrophy in BLA bipolar neurons			[50]

12	CIS	2 h/d for 10 days	Male Wistar rats	*↔* dendrites in CeA ↑ dendrites in BNST			[125]

13	CIS	2 h/d for 10 days	Male Wistar rats	↑ dendritic length in BLA			[126]

14	CIS	2 h/d for 10 days	Male Wistar rats	↑ spine density in the BLA			[127]

15	CIS	2 h/d for 21 days	Male Wistar rats	↑ dendritic arborization BLA, ↑ spine density ↑ synaptic connectivity	↑ anxiety-like behavior		[128]

16	CIS	2 h/day for 10 days	Male Wistar rats	↑ spine density in BLA LTP ↑ (thalamic-LA)	sIPSC frequency ↓		[129]

17	AIS	2 h	Male Wistar rats	*↔* spine density or dendritic arborization 1 d later, ↑ spine density 10 d later in BLA			[127]

18	CUMS	8 weeks	Adult male SD rats	↑ synaptic length of the active zone in BLA ↑ PSD thickness in BLA	↑ synaptic proteins are correlated with depression-like behaviors	↓ PSD93, *↔* PSD95, and *↔* spinophilin *↔* synapsin *↔* synaptophysin	[52]

19	CUMS	14 days	Male Swiss albino mice	↑ spine density in BLA ↑ dendritic length in BLA	Associated with depression-like behaviors		[51]

20	Chronic CORT	20 days	C57BL/6 mice	↑ spine density in BLA, recovery to normal level with a washout period			[130]

21	CORT drinking water	50 *μ*g/mL for 14 days	Adult male SD rats		↑ GluR1 and synaptophysin in the LA	↑ IEGs Arc/Arg3.1 and Egr-1 in the LA	[131]

22	Single prolonged stress	2 h restraint, 20 min forced swimming	Adult male SD rats	↑ dendritic arborization in BLA *↔* in CeA neurons		↑ NPY *↔* CaMKII and MR/GR expression in the BLA	[132]

23	Single elevated platform acute stress	30 min, single	Male SD rats	↑ total spine density ↑ mushroom spine density in BLA; ↓ number and the length of branches in BLA			[133]

24	Chronic social instability stress	1 h/day for 35 days	Adolescent 28-day-old SD rat	↓ spine density in BLA		↑ truncated TrkB, ↓ full-length TrkB and SNAP-25	[134]
			Adult, 56-day-old SD rat	↑ spine density in BLA		↑ full-length and truncated TrkB	

CRS: chronic restraint stress. CUMS: chronic unpredictable mild stress. CIS: chronic immobilization stress. AIS: acute immobilization stress. BA: the basal nucleus of the amygdala. BLA: the basolateral amygdala. LA: the lateral nucleus of the amygdala. MePD: posterodorsal medial amygdala. eCB: endocannabinoid. 2-AG: eCB 2-arachidonoylglycerol. MAGL: monoacylglycerol lipase, an enzyme for degrading 2-AG. CORT: corticosterone. Tianeptine: an antidepressant. *↔*: no change. ↓: decrease. ↑: increase.

**Table 4:** The effects of chronic stress on dendritic spines on nucleus accumbens (NAc).

#	Stress	Paradigms	Animals	NAc	Function	Proteins or mRNA	References
1	CRS	10 days	Male D1R and D2R mice	↓ AMPAR/NMDAR ratio in D1R-MSNs via MC4R; induces LTD in D1R-MSN	MC4R activation and LTD in NAc are required for stress-induced anhedonia	MC4Rs, **α** **-MSH**	[135]

2	CRS	1 h/day for 14 days	Male SD rats	Chronic cannabinoid exposure prevents impaired memory via CB1	CB1/2 receptor agonist prevents CRS-induced-impairment LTP in NAc and in the spatial task	↓ glucocorticoid receptors in the Amg, NAc, PFC, and hippocampus	[121]

3	CUMS	3 stressors/day for 21 days	Male Wistar rats	↑ neuron density in DMS; ↓ neuron density in DLS; ↑ dendritic length in DLS; *↔* spine density in DS			[107]

4	CSDS	10 min/day for 10 days	Male C57/BL6J and CD1		↑ ΔFosB induced by CSDS is required for resilience	↑ ΔFosB in resilience mice	[136]

5	CSDS	10 min/day for 10 days	Male C57/BL6 and CD1		↓ fEPSP in NAc; disrupted NMDAR-dependent LTD in cortico-NAc	↓ NR2B surface and PSD95 in NAc; *↔* NR2A, Syn and NR1	[137]

6	CSDS	5 min/each total 3 times	C57BL/6J and CD1 mice	↑ I*κ*K activity ↑ thin spine density in MSNs	I*κ*K enhances social avoidance behavior	↑ inhibitor of *κ*B kinase (I*κ*K)	[138]

7	CSDS	10 min/day for 10 days	C57BL6/J and CD1 mice	↑ stubby spine density in MSNs in susceptible mice	↑ frequency of mEPSCs in NAc of susceptible mice	↑ I*κ*B kinase (I*κ*K) in NAc in susceptible mice	[139]

8	CSDS	10 min/day for 10 days	C57BL6/J and CD1 mice	↑ Dnmt3a levels in NAc ↑ spine density in NAc	Dnmt3a regulates depression-like behaviors	↑ DNA methyltransferases (Dnmt3a)	[140]

9	CSDS	10 min/day for 10 days	Male C57/BL6 and CD1	↑ stubby spine density in MSNs in susceptible mice in a Rac-1-dependent manner	↑ cofilin puncta colocalization with stubby spines	↓ Rac-1 mRNA levels in NAc of susceptible mice ↓ Rac-1 mRNA levels in NAc of depressed patients	[141]

10	CSDS	10 min/day for 10 days	Male C57/BL6 and CD1	Excitatory transmission at ILT-NAc MSN synapses controls susceptibility to CSDS	↑ AMPAR/NMDAR ratio only at ILT inputs to MSNs of susceptible mice	↑ Vglut22, but not Vglut1 in MSN of susceptible mice	[142]

11	CSDS	10 min/day for 10 days	Male C57/BL6 and CD1	↑ uEPSC amplitude in D1R ↓ uEPSC amplitude in D2R mushroom, not thin spines in NAC MSNs in resilient, but not susceptible mice	CSDS does affect uEPSC amplitude mushroom or thin spines of D1-MSNs or D2-MSNs in susceptible mice		[143]

12	CSDS	10 min/day for 10 days	C57BL6/J and CD1 mice	DNA microarrays; some genes specific to susceptibility in VTA and NAC are identified in susceptible mice	↑ firing in VTA DA neurons in susceptible mice	↑ BDNF, Akt, GSK-3*β*, and ERK1/2 in NAc of susceptible mice	[144]

13	CSDS	10 min/day for 3 days	Male C57/bl6 and CD1	↓ sIPSC frequency in NAc in control, not stressed mice	↑ sensitivity of striatal GABA synapses to the stimulation of cannabinoid CB1R	CB1R ↓	[145]

14	CSDS	10 min/day for 10 days	Male C57/bl6 and CD1	↑ vHIP-NAc synaptic transmission is prosusceptible ↓ LTD of vHIP-NAc synaptic transmission is proresilient	vHIP afferents to NAc uniquely regulate susceptibility to CSDS ↓ activity in vHIP in mice resilient to CSDS		[146]

15	Emotional (ES) and physical stress (PS)	10 min/day for 10 days	Male C57/bl6 adolescent (P35) or adult (P56) and CD1	↑ spine density in NAc in adolescents by ES and PS ↑ spine density in NAc in adult only by PS		ES and PS ↓ p-ERK2 in adolescents but ↑ p-ERK2 in adult	[147]

16	Prenatal stress		Male and female rats	↑ spine density in NAc			[148]

Syn: synaptophysin. CRS: chronic restraint stress. CUMS: chronic unpredictable mild stress. CIS: chronic immobilization stress. CSDS: chronic social defeat stress. MC4R: melanocortin 4 receptor. DMS: dorsal medium striatum. DLM: dorsal lateral striatum. DS: dorsal striatum. ILT: intralaminar thalamus. MSNs: medium spiny neurons. NAc: nucleus accumbens. vHIP: ventral hippocampus. uEPSCs: unitary excitatory postsynaptic currents. *↔*: no change. ↓: decrease. ↑: increase.
